# Comparison of Efficacy and Ocular Surface Disease Index Score between Bimatoprost, Latanoprost, Travoprost, and Tafluprost in Glaucoma Patients

**DOI:** 10.1155/2018/1319628

**Published:** 2018-03-07

**Authors:** Wissam Georges El Hajj Moussa, Rebecca Georges Farhat, Joseph Claud Nehme, Marwan Antoun Sahyoun, Alexandre Raymond Schakal, Alexandre Edmond Jalkh, Mariana Pierre Abi Karam, Georges Georges Azar

**Affiliations:** ^1^Eye and Ear Hospital International, Naccache, Lebanon; ^2^Holy Spirit University, Kaslik, Lebanon; ^3^Saint Joseph University, Beirut, Lebanon

## Abstract

**Aim:**

The purpose of this study is to evaluate and compare the efficacy of 4 prostaglandin analogues (PGAs) and to determine the incidence of ocular surface disease in newly diagnosed, primary open-angle glaucoma (POAG) patients started on one of those 4 PGAs: bimatoprost (benzalkonium chloride, BAK, 0.3 mg/mL), latanoprost (BAK 0.2 mg/mL), travoprost (polyquad), and tafluprost (BAK-free).

**Patients and Methods:**

In this single-center, open-label trial, 32 patients newly diagnosed with POAG were randomly started on one of the four PGAs. All patients underwent a complete ophthalmological exam at presentation and at 1, 3, and 6 months of follow-up. Dry eye disease (DED) was assessed using the original Ocular Surface Disease Index (OSDI) questionnaire, in order to evaluate the impact of the drops on the quality of life of patients.

**Results:**

The mean age was 60.06 years ± 11.76. All four drugs equally and significantly reduced the intraocular pressure (IOP) with respect to the baseline IOP. There was a trend for a slightly greater reduction of IOP with bimatoprost, but the difference was not found to be statistically significant when compared to other PGAs. OSDI scores were significantly superior for travoprost (10.68 ± 5.73) compared to the other three drugs (*p* < 0.05). Latanoprost caused the most significant eyelash growth and iris discoloration. Conjunctival hyperemia and superficial keratitis occurrence were similar in the four groups.

**Conclusion:**

All prostaglandin analogues equally and significantly reduce the IOP in patients with POAG. According to the results of the OSDI score, latanoprost seems to be the least tolerated among the four drugs.

## 1. Introduction

Glaucoma is a chronic and degenerative ophthalmic pathology characterized by a group of specific injuries to the optic nerve head. It is the second preventable leading cause of blindness, after cataract, with around 60.5 million cases reported in 2010 [1] and 80 million cases estimated to ensue in 2020. Primary open-angle glaucoma (POAG) is the most common form of this disease. The intraocular pressure (IOP) is considered to be the only modifiable risk factor for POAG, and its control significantly decreases the progression rate of POAG [2]. As a result, most of the available treatment strategies (drops, laser, and surgery) aim at reducing the IOP. The medical treatments are the most common initial management methods.

Prostaglandin analogs (PGAs) have become the first-line therapeutic class for medical treatment of glaucoma worldwide. Their efficacy as a monotherapy and in combination with other agents, their generally well-tolerated systemic safety profile, and their convenient once-daily dosing contributed to their popularity [3]. Nevertheless, PGAs commonly cause ocular adverse effects, such as conjunctival hyperemia, keratitis, follicular conjunctivitis, iris and periocular skin pigmentation, eyelash growth, and herpes reactivation [4]. A part of these side effects is attributed to the preservatives used in conjunction with the PGA molecule. The impact of preservatives on the ocular surface has gained more attention over the years; hence, other preservative molecules have been used, and novel preservative-free PGAs have been developed.

Benzalkonium chloride (BAK) is the most commonly used preservative in glaucoma preparations. BAK is a proinflammatory and proapoptotic molecule which can damage the tear film by emulsification of the lipid layer [4, 5]. Polyquaternium, a preservative also used in glaucoma medications, is known to have less corneal toxicity, as well as less rupture of cellular junctions, when compared to BAK [6].

The currently prescribed PGAs in our region are bimatoprost 0.01% (with BAK 0.02%), latanoprost 0.005% (with BAK 0.02%), travoprost 0.004% (with 0.001% polyquad), and tafluprost 0.0015% (preservative-free). The latter is the newest addition to the market.

Each PGA has been compared to the gold standard medication, timolol, in randomized clinical trials in order to evaluate the capacity to lower the IOP [7–10]. Head-to-head studies compared the efficacy and tolerability of some PGAs [11–14]; however, no previous trial has concomitantly included the 4 agents in patients naïve to antiglaucomatous therapy. Remarkably, earlier studies and reviews reported conflicting results on their tolerability and relative ocular hypotensive efficacy [15–21].

## 2. Materials and Methods

### 2.1. Study Design and Participants

This is a single-center, prospective, open-label study, conducted between June 2015 and December 2016 at Eye and Ear Hospital International, Naccache, Lebanon. The aim of this study is to compare the efficacy and tolerability of the four PGAs: bimatoprost (benzalkonium chloride, BAK 0.3 mg/mL), latanoprost (BAK 0.2 mg/mL), travoprost (polyquad), and tafluprost (BAK-free). The PGA was prescribed daily for 6 months in patients newly diagnosed with POAG. The approval to conduct this study was obtained by the Research and Development department along with the institutional review board of the Eye and Ear Hospital International. The study adhered to the tenets of the Declaration of Helsinki.

Recruited patients were 18 years old and above, newly diagnosed with POAG, naïve to antiglaucomatous drugs, whose IOP was controllable by monotherapy, and who did not present any ocular surface disease (including anterior or posterior blepharitis, keratitis, ocular dryness, and follicular or papillary conjunctivitis) or any other ophthalmic pathology at the time of inclusion.

The exclusion criteria were age less than 18 years, ocular surface findings at baseline, presence of any associated ophthalmic pathology, concomitant use of drugs affecting the ocular surface, advanced glaucoma uncontrolled by monotherapy, and any history of uveitis or cataract extraction surgery within one month prior to the day of inclusion.

### 2.2. Treatment and Assessments

Included patients were randomly assigned to receive one of the four PGAs: bimatoprost 0.01% (with BAK 0.02%), latanoprost 0.005% (with BAK 0.02%), travoprost 0.004% (with 0.001% polyquad), and tafluprost 0.0015% (preservative-free). Patients were instructed to instill one drop in each eye every evening between 7 : 00 pm and 9 : 00 pm, starting on the day of the first visit. Follow-up visits were scheduled at months 1, 3, and 6. The IOP was measured using the Goldmann applanation tonometer, between 8 : 00 am and 10 : 00 am. It was recorded at every visit. The IOP, a main endpoint in this study, was measured two times consecutively by two glaucoma surgeons (GA and RF) with masked values. Then, the average of the two pressures was obtained. Tolerability was evaluated objectively on the clinical exam and subjectively by means of the Ocular Surface Disease Index (OSDI score). Recruited patients underwent a complete bilateral and comparative ophthalmologic examination including measurement of the best corrected visual acuity (BCVA), slit-lamp examination for a detailed visualization of the ocular surface (Schirmer test, tear breakup time (TBUT), and fluorescein test), and fundus exam assessment after pupillary dilation with 1% tropicamide. The evaluation of the iris pigmentation was based on the basic iris color classification of the eye.

The same examination was repeated at 1, 3, and 6 months of follow-up for every subject in this study. The elements looked for at each visit were the presence of macroscopic conjunctival hyperemia graded by gross inspection in comparison with standard photographs, superficial punctiform keratitis (using the Oxford classification), follicular conjunctivitis, iris pigmentation using photographic iris color, eyelash growth, and herpes reactivation.

Patients with pressures remaining above the cut-off IOP, set at 20 mmHg, despite one month of treatment, and who required a second class of antiglaucoma medication, were excluded from the analysis. Patients who developed severe acute side effects such as sever allergy and angioedema were also withdrawn from the study due to the obligate cessation of the PGAs.

During the sixth-month follow-up visit, the PGA tolerability was assessed subjectively on the basis of the OSDI score. The OSDI is evaluated on a scale from 0 to 100 where the score given is directly proportional to the severity of the symptoms. The ocular surface injury is classified as normal (0–12), minimal [13–22], moderate (23–32), and severe (33–100).

### 2.3. Data Analysis

All the collected information was regrouped in a data base program on a personal computer. Statistical analysis was performed using commercially available software (SPSS Version 20.0, SPSS Inc., Chicago, Illinois). Continuous variables were noted by means and their corresponding standard deviations (SD). Categorical data was presented by frequencies and percentages. The statistical analysis of the continuous and categorical data depended on the Student's *t*-test and the chi-square test, respectively. Assuming a normal distribution of the data, ANOVA *F*-test, followed by the Dunnett's post hoc test, was used to compare the four small size samples. Statistical significance was set at *p* values of <0.05 at 95% confidence levels.

## 3. Results

### 3.1. Demographic Characteristics

Forty patients were randomized to receive one of the four PGAs. Fifty percent of patients were women. The mean age was 60 years ± 11.76 SD (range, 38–82). In 6 patients, PGA monotherapy was not sufficient to achieve a posttreatment IOP less than 20 mmHg (1 patient on bimatoprost, 2 patients on latanoprost, 2 patients on travoprost, and 1 patient on tafluprost). Therefore, they were withdrawn from the study. Two other patients (1 patient on bimatoprost and 1 patient on latanoprost) were lost to follow-up. In total, 32 patients (80%) completed the study: Eight patients (25%) were started on bimatoprost, 7 patients (21.9%) on latanoprost, 8 patients (25%) on travoprost, and 9 patients (28.1%) on tafluprost. Patients' demographic and baseline characteristics are similar among the four treatment groups (Table 1).

### 3.2. Mean Intraocular Pressure

**Figure 1:**
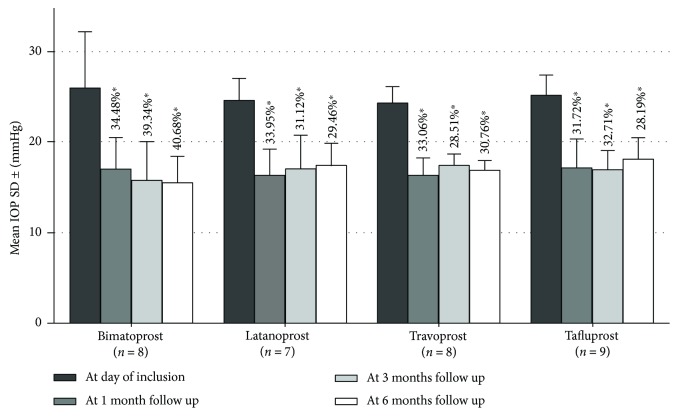
Mean IOP in POAG patients treated with different prostaglandins in monotherapy, at inclusion and at 1 month, 3 months, and 6 months of follow-up. The percentage of IOP reduction is written above each column. A *p* value <0.05 is marked by an asterisk. IOP: intraocular pressure; POAG: primary open-angle glaucoma.

At the initial visit, the mean IOP for the group treated with bimatoprost was 26.13 mmHg ± 6.15 SD. That of the latanoprost group was 24.71 mmHg ± 2.36 SD and 24.38 mmHg ± 1.85 SD for the travoprost group. The tafluprost group had an initial mean IOP of 25.22 mmHg ± 2.28 SD. The mean IOPs of the bimatoprost group at 1 month (M1), 3 months (M3), and 6 months (M6) of follow-up were 17.12 mmHg ± 3.42 (34.48% reduction), 15.58 mmHg ± 4.20 (39.34% reduction), and 15.50 mmHg ± 2.93 (40.68% reduction), respectively. The latanoprost group mean IOPs and reductions were 16.32 mmHg ± 2.96 (33.95% reduction), 17.02 mmHg ± 3.76 (31.12% reduction), and 17.43 mmHg ± 2.57 (29.46% reduction) at the respective follow-up visits. As for the travoprost group, the values were 16.32 mmHg ± 2.01 (33.06% reduction), 17.43 mmHg ± 1.32 (28.51% reduction), and 16.88 mmHg ± 1.13 (30.76% reduction) corresponding to each visit. Lastly, the group treated with tafluprost presented the mean IOPs at 17.22 mmHg ± 3.09 (31.72% reduction), 16.97 mmHg ± 2.07 (32.71% reduction), and 18.11 mmHg ± 2.42 (28.19% reduction) at the time of follow-up visits (Figure 1). The four treatments prescribed led to a clinically and statistically significant decrease in the IOP at M1, M3, and M6 follow-up visits with respect to baseline measurements (*p* < 0.01).

Despite the trend of greater decrease in IOP seen with the bimatoprost group after the 6-month treatment, this difference was not statistically significant when compared to the other PGAs (*p* = 0.112).

### 3.3. Adverse Effects and Tolerability

The adverse effects of the four agents were evaluated at each visit. Iris discoloration was found in two patients (both on latanoprost) who initially had a homogenous blue-grey shade. During follow-up, there has been an increase in superficial pigmentation of their iris with a “granular” appearance (type 1 change). Eyelash growth occurred in 1 patient on bimatoprost, 4 patients on latanoprost, and 2 patients on travoprost. These two adverse effects were significantly associated with latanoprost in comparison to the groups treated with bimatoprost, travoprost, and tafluprost (*p* = 0.05). Conjunctival hyperemia, superficial keratitis, and follicular conjunctivitis were all reported in the four groups at different proportions, but a significant difference between the four groups was not found. Herpes reactivation, diagnosed on the basis of clinical observation, was only found in one patient who was treated with latanoprost. However, this finding was not statistically significant (Table 2). None of the patients developed prostaglandin-associated orbitopathy or any severe acute side effects (acute allergy and angioedema) during the whole follow-up period.

**Figure 2:**
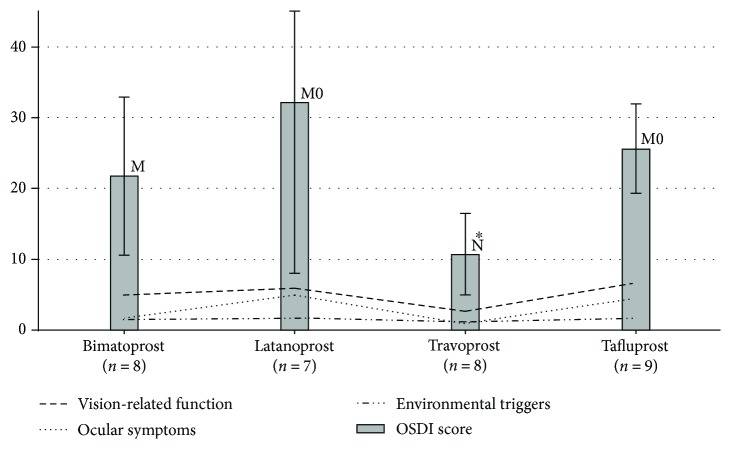
Different OSDI scores of patients treated with different prostaglandins. The three dashed lines represent the three subscales analysed to obtain a final OSDI score: vision-related function, ocular symptoms, and environmental triggers. A *p* value <0.05 is marked by an asterisk. OSDI indicates ocular surface disease index; N, M, and M0 represent, respectively, normal, mild, and moderate eye disease as defined by both physician's assessment and a composite disease severity score; POAG: primary open-angle glaucoma.

Subjective assessment of tolerability of the four PGAs was done using the OSDI score after 6 months of treatment. The scores were 21.76 ± 11.10 for the group treated with bimatoprost, 32.13 ± 24.10 for the latanoprost group, 10.68 ± 5.73 for the travoprost group, and 25.60 ± 6.25 for the tafluprost group (Figure 2).

Statistically, the mean OSDI score of the group treated with travoprost was significantly inferior to the mean OSDI score of each of the three other groups (*p* < 0.05). In other words, travoprost was the most tolerated drug among the four PGAs. Latanoprost had the highest OSDI score (32.13), but its relevance was not statistically significant when compared to the scores of the other PGAs.

## 4. Discussion

Several PGAs are available in the market; hence, the choice of treatment is based on the IOP lowering ability, tolerability, and adverse effects. To the best of our knowledge, our study is the first to compare simultaneously the four PGAs in patients newly diagnosed with POAG, who did not receive any previous antiglaucomatous treatment and were clear from any ocular surface disease at presentation.

The results of this comparative prospective study demonstrate that the four PGAs are equally effective in reducing IOP in patients with POAG, findings similar to those in the literature [7–10].

When comparing the four PGAs, all drugs equally and significantly reduced the IOP from baseline. Despite the slight trend for a greater reduction of IOP with bimatoprost, this finding was not statistically significant when compared to other PGAs. These results are in concordance with many studies in the literature. For instance, Ranno et al. studied POAG patients previously treated with bimatoprost, latanoprost, and travoprost for a minimum of 3 months and then switched to tafluprost. They noted the absence of any differences in the mean IOP with tafluprost compared to latanoprost and travoprost [22]. Our results are also comparable to those of other studies revealing that latanoprost, bimatoprost, and travoprost have similar efficacy [19]. However, those conclusions are contradicted in some literature reviews. In fact, a meta-analysis published in 2014 found that bimatoprost had a greater efficacy, followed by travoprost, latanoprost, and tafluprost [23]. Another study by Hommer and Kimmich showed that tafluprost had a greater efficacy when compared to the other three groups by providing further IOP reduction after switching to this molecule [24]. The tolerability of locally used drugs is a key factor in treating chronic diseases such as glaucoma. Two aspects are important in this context: the preservative component of the medication and the molecule itself. Existing evidence shows that long-term use of drugs containing BAK causes severe dryness, inflammatory cell infiltration, blepharitis, superficial punctuate keratitis, and eyelid eczema [4, 5, 25–27]. The polyquad preservative is less toxic to the ocular surface when compared to BAK. In this study, we evaluated the tolerability based on the OSDI score. Travoprost was shown to have the lowest score, hence a better tolerance; while latanoprost had the highest score. The difference in the results was otherwise not statistically significant. These findings were in part predictable. Travoprost was expected to be more tolerable than BAK-containing bimatoprost and latanoprost since the former contains polyquad. This notion is confirmed by a recent study based on electronic microscopy, histological examination of the cornea, and biomolecular exams. The author concluded that products containing BAK induce an important damage to the cornea [28]. However, those findings are also debatable. Other studies compared bimatoprost with and without BAK and concluded that the two treatments were similar in efficacy and tolerability, rejecting therefore the findings about BAK toxicity and its utility for better bioavailability of the drug [22].

An interesting finding was the higher tolerance of polyquad-based travoprost when compared to preservative-free (PF) tafluprost in our series. This suggests that local damage of the ocular surface could be induced by the direct effects of the drug, independently of the preservative product. However, those results are contradictory in the literature: while some studies confirm a “nonharmful” effect of polyquad and a similar toxicity between polyquad-containing products and PF products, other studies confirm a better tolerability for PF tafluprost [22, 24].

Slit-lamp examination provided an objective evaluation of tolerability. Our finding that hyperemia, the most common side effect of PGA, is associated with all four drugs without statistically significant difference contradicts the results of the meta-analysis that attributes hyperemia to bimatoprost [23]. We also found a significant association between the use of latanoprost and iris pigmentation as well as eyelash growth. One case of herpes reactivation was noted in the group of patients receiving latanoprost. Although these results are not significant in our study, similar cases are also reported in the literature [29–31].

Our study is limited by the small number of patients due to the strict exclusion criteria and the short recruitment period. The efficacy and tolerability analyses were studied over a short period of time; therefore, the long-term efficacy and tolerability of the treatment were not considered. On the other hand, our study was not a double-blinded study; neither the patients nor the physicians were blinded to the drug used.

In conclusion, this study demonstrated that all four PGAs reduce equally and effectively the IOP, with bimatoprost being nonsignificantly more effective. Travoprost was found to be the most tolerated agent and even superior to PF agent. Latanoprost was more associated with iris pigmentation and eyelash growth.

## Figures and Tables

**Table 1:** Demographic and baseline characteristics of all primary open-angle glaucoma patients controllable by prostaglandin (PG) monotherapy.

	Bimatoprost (*n* = 8)	Latanoprost (*n* = 7)	Travoprost (*n* = 8)	Tafluprost (*n* = 9)	*p* value
Mean age, years (mean ± SD)	62.75 ± 14.16	58.71 ± 7.09	61.75 ± 16.29	57.22 ± 8.33	0.77
Sex, male (%)	4 (50.0)	6 (85.7)	3 (3.75)	3 (33.3)	0.18
Mean intraocular pressure at inclusion, mmHg (mean ± SD)	26.13 ± 6.15	24.71 ± 2.36	24.38 ± 1.85	25.22 ± 2.28	0. 79

**Table 2:** Comparison of topical adverse effects of the four prostaglandins in primary open-angle glaucoma patients controlled by monotherapy.

	Bimatoprost (*n* = 8)	Latanoprost (*n* = 7)	Travoprost (*n* = 8)	Tafluprost (*n* = 9)	*p* value
Adverse effect, *n* (%)					
Conjunctival hyperemia	6 (75.0)	5 (71.4)	4 (50.0)	9 (100.0)	0.13
Superficial keratitis	4 (50.0)	4 (57.1)	3 (3.8)	3 (33.3)	0.76
Follicular conjunctivitis	2 (25.0)	4 (57.1)	3 (3.8)	5 (55.5)	0.51
Iris hyperpigmentation	0 (0)	2 (28.6)	0 (0)	0 (0)	0.05
Eyelash growth	1 (12.5)	4 (57.1)	2 (25.0)	0 (0)	0.05
Herpetic reactivation	0 (0)	1 (14.3)	0 (0)	0 (0)	0.30
